# NSC109268 potentiates cisplatin-induced cell death in a p53-independent manner

**DOI:** 10.1186/1750-2187-5-4

**Published:** 2010-05-10

**Authors:** Eswar Shankar, Chandreyi Basu, Brett Adkins, Wolfram Siede, Alakananda Basu

**Affiliations:** 1 Department of Molecular Biology and Immunology, University of North Texas, Health Science Center, Fort Worth, Texas, USA; 2 Institute for Cancer Research, University of North Texas Health Science Center, Fort Worth, Texas, USA; 3 Department of Cell Biology and Anatomy, University of North Texas Health Science Center, Fort Worth, Texas, USA; 4 Current address: Department of Urology, Case Western Reserve University, Cleveland, OH 44106, USA

## Abstract

**Background:**

Ovarian cancer is the leading cause of death among gynecological cancers. Cisplatin is one of the most effective anticancer drugs used in the treatment of ovarian cancer. Development of resistance to cisplatin limits its therapeutic use. Most of the anticancer drugs, including cisplatin, are believed to kill cancer cells by inducing apoptosis and a defect in apoptotic signaling can contribute to drug resistance. The tumor suppressor protein p53 plays a critical role in DNA damage-induced apoptosis. During a yeast-based drug screening, NSC109268 was identified to enhance cellular sensitivity to cisplatin. The objective of the present study is to determine if p53 is responsible for cisplatin sensitization by NSC109268.

**Results:**

NSC109268 enhanced sensitivity of ovarian cancer 2008 cells and its cisplatin resistant counterpart 2008/C13* cells which express wild-type p53. The potentiation of cisplatin sensitivity by NSC109268 was greater in 2008/C13* cells compared to 2008 cells. Cisplatin caused a concentration-dependent increase in p53 in 2008 and 2008/C13* cells, and the induction of p53 correlated with cisplatin-induced apoptosis as determined by the cleavage of PARP. NSC109268 alone had no effect on p53 but it enhanced p53 level in response to cisplatin. Knockdown of p53 by siRNA, however, did not attenuate cell death in response to cisplatin or combination of NSC109268 and cisplatin.

**Conclusions:**

These results demonstrate that NSC109268 enhances sensitivity of ovarian cancer 2008 cells to cisplatin independent of p53.

## Background

*cis*-Diamminedichloroplatinum(II) or cisplatin is one of the most important anticancer drugs used in the treatment of solid tumors, especially ovarian, testicular, cervical and small cell lung carcinomas. Dose-limiting toxicity to normal tissues and acquisition of resistance by tumor tissues to cisplatin, however, poses a significant problem in cisplatin therapy. Identification of agents that can sensitize tumor cells to cisplatin, and circumvent or prevent cisplatin resistance should have significant impact in cisplatin-based therapy.

The anticancer activity of cisplatin is believed to be due to its interaction with chromosomal DNA [1]. However, only a small fraction of cisplatin actually interacts with DNA, and inhibition of DNA replication cannot solely account for its biological activity [2]. The effectiveness of anticancer agents depends not only on their ability to induce DNA damage but also on the cell's ability to detect and respond to DNA damage [3,4]. The tumor suppressor protein p53 plays a critical role in DNA damage signaling [5]. It is activated in response to DNA damage and triggers transcription of genes involved in cell cycle, apoptosis, senescence and DNA repair [6,7]. The *p53 *gene is mutated in 50% of human cancers, and it is often inactivated by oncogenic viruses in those cases in which *p53 *is not mutated [8-10]. For example, the majority of cervical cancer cells contain wild-type *p53 *but the E6 gene product of human papilloma virus (HPV) results in the rapid degradation of p53 through the ubiquitin proteasome pathway [10]. Thus, these cells have the same functional consequences as a mutated *p53 *gene.

NSC109268 was found to enhance sensitivity of budding yeast *Saccharomyces cerevisiae *during a novel yeast-based genetic screening of the Diversity Set compound library provided by the Developmental Therapeutics Division (NCI). It also increased sensitivity of human cancer cells to cisplatin [11]. In the present study, we have examined the ability of NSC109268 to sensitize parental and cisplatin-resistant variants of p53-positive ovarian carcinoma 2008 and p53-null human cervical carcinoma HeLa cells to cisplatin. NSC109268 enhanced sensitivity of human ovarian carcinoma 2008 cells to cisplatin but it had no effect on the sensitivity of HeLa cells. However, the mechanism of cisplatin sensitization by NSC109268 did not involve p53.

## Results

### Effect of NSC109268 on cisplatin sensitivity

**Figure 1:**
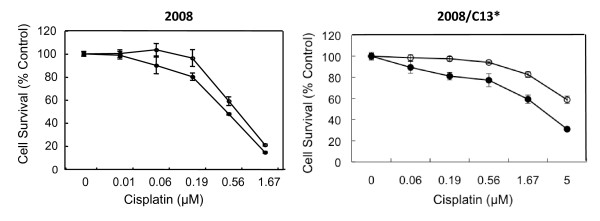
The effects of NSC109268 on the sensitivity of parental (2008) and cisplatin-resistant (2008/C13*) ovarian cancer cells to cisplatin. Cells were treated without (open circle) or with 2 μM NSC109268 (black circle) and varying concentrations of cisplatin for 72 h. Cell survival was determined by the MTT assay. The control values were based upon the results obtained in the absence of any drug but in the presence of vehicle or 2 μM NSC109268. Each value represents the mean ± SD of four determinants from a single microtiter plate. The results are representative of three independent experiments.

**Figure 2:**
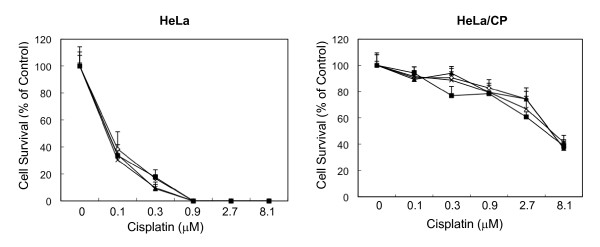
**The effects of NSC109268 on the sensitivity of parental (HeLa) and cisplatin-resistant (HeLa/CP) human cervical carcinoma cells to cisplatin**. Cells were treated without (open circle) or with 1 μM (black square), 2 μM (black triangle) or 3 μM (black circle) NSC109268 and varying concentrations of cisplatin for 72 h. Cell survival was determined by the MTT assay. The control values were based upon the results obtained in the absence of any drug but in the presence of vehicle or NSC109268. Each value represents the mean ± SD of four determinants from a single microtiter plate. The results are representative of three independent experiments.

We compared the ability of NSC109268 to sensitize human ovarian cancer 2008 and human cervical cancer HeLa cells and their cisplatin-resistant counterparts HeLa/CP and 2008/C13* cells, respectively. Figure 1 shows that NSC109268 enhanced the sensitivity of both parental 2008 cells and cisplatin-resistant variant 2008/C13* cells to cisplatin although the effect was more pronounced with 2008/C13* cells. When 2008 cells were treated with different concentrations of cisplatin alone for 72 h, the IC_50 _for cisplatin was 0.8 μM and it decreased to 0.5 μM when treated with both NSC109268 and cisplatin. The IC_50 _of 2008/C13* cells for cisplatin was greater than 10 μM and decreased to 2.8 μM when NSC109268 was included with cisplatin. In contrast, NSC109268 did not influence the sensitivity of parental HeLa and cisplatin-resistant variant HeLa/CP cells to cisplatin (Figure 2). Thus, NSC109268 enhanced the sensitivity of p53-positive ovarian cancer 2008 cells, and it had greater effect on cisplatin-resistant variant compared to parental drug-sensitive cells.

### Effect of NSC109268 on cisplatin-induced p53 level

**Figure 3:**
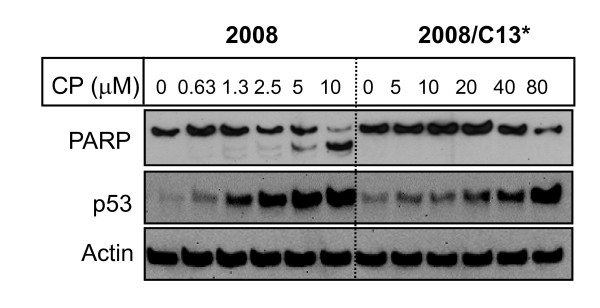
**Effect of cisplatin on induction of p53 and cleavage of PARP**. Cells were treated with indicated concentrations of cisplatin for 24 h. Western blot analysis was performed with total cell lysates using indicated antibodies. Actin was used to control for loading differences.

Since p53 plays a critical role in DNA damage signaling, we compared the effects of cisplatin on the cleavage of PARP and p53 induction in 2008 and 2008/C13* cells. Figure 3 shows that cisplatin caused a concentration-dependent increase in PARP cleavage in 2008 cells but not in 2008/C13* cells. Cisplatin also caused a concentration-dependent increase in p53 level in both 2008 and 2008/C13* cells but much higher concentrations of cisplatin were required to induce p53 in 2008/C13* cells compared to 2008 cells. Thus, the induction of p53 by cisplatin was associated with an increase in cisplatin-induced apoptosis in 2008 cells as determined by the cleavage of PARP. 2008/C13* cells were resistant to cisplatin-induced apoptosis.

**Figure 4:**
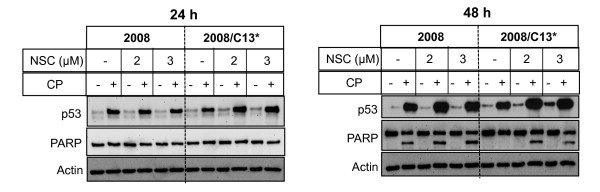
**Effect of NSC on p53 induction and cleavage of PARP**. Cells were treated with or without 2 or 3 μM NSC109268 and 1 μM (2008) or 40 μM cisplatin (2008/C13*) for 24 h or 48 h. Western blot analysis was performed with total cell lysates using indicated antibodies. Actin was used to control for loading differences.

To determine if NSC109268 sensitizes these ovarian carcinoma cells to cisplatin via p53-dependent mechanisms, we treated 2008 and 2008/C13* cells with the combination of NSC and cisplatin, and monitored p53 level and PARP cleavage. In this experiment, we treated 2008 and 2008/C13* cells with 1 μM and 40 μM cisplatin, respectively that had no effect on PARP cleavage at 24 h (Figure 3). As shown in Figure 4, cisplatin caused a substantial increase in p53 in both 2008 and 2008/C13* cells but NSC109268 alone had little effect on p53 induction. A 24 h treatment with NSC109268 and cisplatin caused a modest increase in p53 in 2008/C13* cells but not in 2008 cells. A 48 h treatment with NSC109268 and cisplatin increased p53 in both 2008 and 2008/C13* cells but the effect was more pronounced in 2008/C13* cells. A 24 h treatment with cisplatin had little effect on PARP cleavage even when coincubated with 2 μM and 3 μM NSC109268. A 48 h treatment of 2008 cells with 1 μM cisplatin caused PARP cleavage, which increased modestly by the inclusion of NSC109268. While 48 h treatment of 2008/C13* cells with 40 μM cisplatin alone had no effect on the cleavage of PARP, the combination of NSC109268 and cisplatin induced cleavage of PARP in 2008/C13* cells.

**Figure 5:**
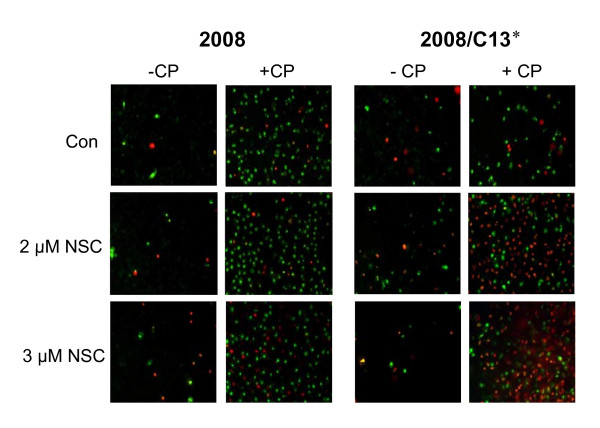
**Effect of NSC on cisplatin-induced cell death**. Cells were treated with or without 2 or 3 μM NSC109268 and 1 μM (2008) or 40 μM cisplatin (2008/C13*) for 48 h. Following incubation, cells were stained with Yo-Pro-1 and PI as described in the Methods. Fluorescent staining was visualized using Zeiss Axiovert 40 inverted microscope.

We also examined the effect of NSC109268 on cisplatin-induced cell death by staining cells with Yo-Pro-1 and propidium iodide (PI). The green-fluorescent dye Yo-Pro-1 can enter apoptotic cells, whereas the red fluorescent dye PI can only enter dead cells. Thus, when cells are stained with both Yo-Pro-1 and PI, apoptotic cells show green fluorescence whereas dead cells show primarily red fluorescence and some green fluorescence. As shown in Figure 5, NSC109268 enhanced cisplatin-induced cell death in 2008/C13* cells but had only a modest effect on cisplatin-induced cell death in 2008 cells.

### Effect of p53 knockdown on the sensitization of cisplatin-induced cell death in 2008/13* cells

**Figure 6:**
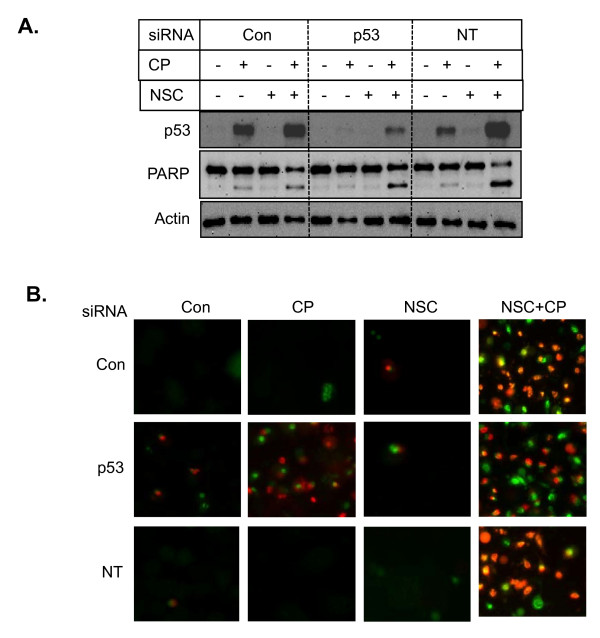
**Effect of p53 knockdown on cisplatin sensitization by NSC109268**. Cells were transfected with or without control non-targeting siRNA or p53 siRNA as described under Methods. Cells were then treated with or without 2 μM NSC102968 and 40 μM cisplatin. A, Western blot analysis was performed with total cell lysates as described under legend to Figure 4. B, Cells were stained with Yo-Pro-1 and PI as described under legend to Figure 5.

To determine if potentiation of cisplatin-induced cell death by NSC109268 was due to upregulation of p53 in 2008/13* cells, we examined the consequence of p53 depletion on the effects of NSC109268 on PARP cleavage and cell death triggered by cisplatin. Figure 6A shows that knockdown of p53 by siRNA effectively depleted p53 and little p53 could be detected following treatment with cisplatin. p53 was induced following treatment with NSC109268 and cisplatin even when cells were transfected with p53 siRNA albeit at much lower level compared to cells transfected without or with control siRNA. However, knockdown of p53 had little effect on cisplatin sensitivity or potentiation of cisplatin-induced PARP cleavage.

We also monitored cell death by staining cells with Yo-Pro-1 and PI staining (Figure 6B). Knockdown of p53 failed to prevent cisplatin-induced cell death in 2008/C13* cells. If anything, p53 depletion caused a slight increase in cisplatin-induced cell death. Moreover, p53 knockdown had no effect on the potentiation of cisplatin-induced cell death by NSC109268. Thus, NSC109268 enhanced cisplatin-induced cell death via p53-independent pathway.

## Discussion

Cisplatin has been very effective for the treatment of gynecological cancers, such as ovarian and cervical cancers. However, the development of resistance in initially responsive tumors to cisplatin is a major obstacle in cisplatin-based therapy. We inadvertently discovered that NSC109268 sensitizes budding yeast *Saccharomyces cerevisiae *to cisplatin [11]. The results of our present study demonstrate that cisplatin-resistant ovarian cancer 2008/C13* cells are exquisitely sensitive to combined treatment with NSC109268 and cisplatin. We have utilized three independent methods to assess the effects of NSC109268 on cisplatin sensitivity-MTT assay, PARP cleavage and Yo-Pro-1/PI staining. Using all three assays, we have found that NSC109268 had a greater effect in augmenting the sensitivity of cisplatin-resistant 2008/C13* cells to cisplatin compared to its drug-sensitive counterpart.

Most of the anticancer drugs kill cancer cells by inducing apoptosis and a defect in apoptotic signaling could contribute to drug resistance. p53 plays a critical role in eliciting cellular responses to DNA damage, and is frequently mutated in ovarian cancers [12,13]. It has been reported that the status of p53 is an important determinant of cisplatin sensitivity in patients with ovarian cancer [14-16] although some studies suggest that cisplatin-induced cell death is independent of the presence of wild-type p53 [17]. An aberration in p53 has also been implicated in cisplatin resistance [11,18-20]. Introduction of wild-type p53 by adenovirus vector sensitized ovarian cancer cells to cisplatin [21-23]. In the present study, we have used ovarian cancer 2008 cells and its cisplatin-resistant variant 2008/C13* cells which contain wild-type p53. Cisplatin caused induction of p53 in both parental and cisplatin-resistant 2008 cells and the increase in p53 correlated with cisplatin-induced apoptosis (Figure 3). In addition, sensitization of 2008/C13* cells by NSC109268 was associated with an increase in p53 level (Figure 4). Furthermore, NSC109268 failed to sensitize parental and cisplatin-resistant HeLa cells in which p53 is degraded by the human papilloma virus E6 (Figure 2). These results are consistent with the notion that NSC109268 sensitizes ovarian cancer 2008 cells to cisplatin via p53-dependent mechanisms. Following observations, however, argue against the involvement of p53 in the potentiation of cisplatin sensitivity by NSC109268.

Although little p53 could be detected in parental HeLa cells, we have found that p53 is detectable in HeLa/CP cells and it is further increased by the treatment with cisplatin [24]. Perhaps low level of cisplatin-induced DNA damage during the selection of HeLa/CP cells stabilizes p53. We have also found that the level of HPV E6 protein, which degrades p53 is less in HeLa/CP cells [24]. To determine if p53 is responsible for cisplatin sensitization by NSC109268, we depleted endogenous p53 by siRNA silencing. Knockdown of p53 had little effect on cell death by cisplatin or combination of NSC109268 and cisplatin (Figures 6A and 6B). Interestingly, depletion of p53 appears to increase cisplatin-induced cell death in 2008/C13* cells (Figure 6B). These results are consistent with earlier reports that inactivation of p53 may enhance sensitivity of cisplatin-resistant ovarian cancer cells to cisplatin [25].

The effect of NSC109268 on cisplatin sensitivity depends both on the concentrations and duration of exposure to these compounds. While a 24 h treatment with 3 μM NSC109268 alone had little effect on cell survival, a 72 h treatment with ≥ 3 μM NSC109268 caused a substantial decrease in cell growth as determined by the MTT assay (data not shown). The effect of NSC109268 on cell growth inhibition was much more pronounced in 2008/C13* cells compared to 2008 cells [11]. In both yeast and ovarian cancer cells, NSC109268 appears to inhibit progression of cells from the S phase to G2/M phase [11]. Thus, cells arrested at the S phase may be more sensitive to cisplatin. Additionally, we have found that in response to cisplatin, a substantial fraction of 2008/C13* cells were stained with the cell impermeable dye propidium iodide. Since these cells are resistant to apoptosis, it is conceivable that NSC109268 increased cisplatin-induced cell death by enhancing cisplatin-induced necrosis via p53-independent pathway.

The mechanisms of cisplatin resistance are multifactorial and include decrease in drug uptake, increase in DNA repair, increase in cellular thiol content, defect in p53 and increase in antiapoptotic signaling [26]. The protein kinase C (PKC) signaling pathway plays an important role in determining cisplatin sensitivity [27,28]. Activators of PKC sensitized both 2008 [28] and HeLa cells [27] to cisplatin. Furthermore cisplatin resistance was associated with increase in novel PKC and decrease in conventional PKC [29,30]. It remains to be seen if NSC109268 influences the PKC signaling pathway.

NSC109268 may also increase cisplatin sensitivity by facilitating its uptake or decreasing repair of cisplatin-induced DNA damage. Although cisplatin is believed to enter cells via passive diffusion, it has been reported that a fraction of cisplatin enters cells via the plasma membrane copper transporter CTR1 [31,32]. Following internalization, both copper and cisplatin were shown to cause downregulation of CTR1 in ovarian cancer cells by the proteasome-mediated pathway [33]. NSC109268 contains two copper ions and it was shown to inhibit 20S proteasome [34]. Since loss of CTR1 can contribute to cisplatin resistance [32], it is conceivable that NSC109268 inhibits degradation of CTR1 by cispatin resulting in higher intracellular levels of cisplatin and consequently increase in cisplatin-induced cell death. Future studies should determine how NSC109268 attenuates cisplatin resistance via p53-independent mechanisms. Nevertheless, the observation that NSC109268 can partially overcome cisplatin resistance is significant. Since NSC compound can be easily synthesized, it could be used as an adjuvant to cisplatin-based therapy.

## Conclusions

The present study demonstrates that NSC109268 enhances the sensitivity of cisplatin-resistant ovarian cancer 2008/C13* cells to cisplatin. While the induction of p53 by cisplatin or combination of NSC109268 and cisplatin correlates with increased cellular sensitivity to cisplatin, knockdown of p53 had no effect on cisplatin sensitization by NSC109268. Thus, NSC109268 potentiates cisplatin sensitivity via p53-independent mechanism(s). NSC109268 can be used in combination with cisplatin to circumvent cisplatin resistance.

## Methods

### Materials

Cisplatin, MTT and monoclonal antibody to actin were purchased from Sigma (St. Louis, MO). Monoclonal antibodies to p53 and GAPDH were from Santa Cruz Biotechnology, Inc. (Santa Cruz, CA), and monoclonal antibodies to PARP and p53 were obtained from Pharmingen (San Diego, CA). siRNA SMARTpool against p53, and non-targeting SMARTpool siRNA were obtained from Dharmacon (Lafayette, CO). Horseradish peroxidase-conjugated goat anti-mouse and donkey anti-rabbit antibodies were obtained from Jackson ImmunoResearch Laboratories, Inc. (West Grove, PA). Polyvinylidene difluoride membrane was from Millipore (Bedford, MA), and enhanced chemiluminescence detection kit was from Amersham (Piscataway, NJ). Lipofectamine 2000 transfection reagent and YO-PRO-1 were obtained from Invitrogen (Carlsbad, CA). Propidium Iodide was purchased from Molecular Probes (Eugene, OR). NSC109268 was provided by the Developmental Therapeutics Branch of the National Cancer Institute, and was also custom-synthesized by Omm Scientifc, Dallas, TX.

### Cell Culture

Human ovarian cancer 2008 cells and cisplatin-resistant variant 2008/C13* cells developed by Dr. Stephen B. Howell were obtained from the University of California San Diego. Cells were maintained in RPMI 1640 supplemented with 5% heat-inactivated FBS and 2 mmol/L glutamine. Human cervical carcinoma HeLa cells and its cisplatin-resistant variants (HeLa/CP) [35] were maintained in DMEM supplemented with 10% heat-inactivated fetal bovine serum and 2 mmol/L glutamine. Cells were kept in a humidified incubator at 37°C with 95% air and 5% CO_2_.

### Assessment of cell viability by MTT Assay

Exponentially growing cells were plated in microtiter plates and incubated at 37°C in 5% CO2. The following day, cells were pretreated with or without NSC109268 and cisplatin as indicated in the text. The number of viable cells was determined using the dye MTT as previously described (5).

### Assesment of cell death by YO-PRO-1/propidium iodide (PI) assay

Cells were labeled with 0.5 μM Yo-Pro-1 and 2 μM PI and incubated at 37°C for 15 min. Labeling of the cells was visualized using a Zeiss Axiovert 40 inverted microscope with the AxioVision Rel 4.6 software (Zeiss, Göttingen, Germany).

### Immunoblot analysis

Equivalent amounts of protein from total cellular extracts were electrophoresed by SDS-PAGE and transferred electrophoretically to a poly(vinylidene difluoride) membrane. Immunoblot analyses were performed as described previously [36].

### Knockdown of p53 by siRNA

Control siRNA or siRNA targeted against p53 were introduced into cells using Lipofectamine 2000 (Invitrogen) and manufacturer's protocol as previously described [37]. Briefly, cells were seeded one day before transfection. Forty-eight hour following siRNA transfection, cells were treated with NSC109268 and cisplatin as indicated in the text and processed for Western blot analysis.

## Competing interests

The authors declare that they have no competing interests.

## Authors' contributions

ES performed Western blot analysis, siRNA knockdown and Yo-Pro-1/PI staining to determine the involvement of p53 in cisplatin sensitization. CB performed MTT assay to compare the effect of NSC109268 on cisplatin sensitization in 2008 and 2008/C13* cells. BA performed similar study with HeLa and HeLa/CP cells. WS first identified NSC109268 during yeast-based genetic screening and provided intellectual input in this collaborative study. AB designed experiments, analysed data and prepared manuscript. All authors read and approved the final manuscript.
